# Deletion of glycerol channel aquaporin-9 (*Aqp9*) impairs long-term blood glucose control in C57BL/6 leptin receptor–deficient (*db/db*) obese mice

**DOI:** 10.14814/phy2.12538

**Published:** 2015-09-28

**Authors:** Peter Spegel, Aakash Chawade, Søren Nielsen, Per Kjellbom, Michael Rützler

**Affiliations:** 1 Unit of Molecular Metabolism, Lund University Diabetes Centre, CRC, Skåne University HospitalMalmö, Sweden; 2 Department of Immunotechnology, Lund UniversityLund, Sweden; 3 Department of Health Science and Technology, Aalborg UniversityAalborg, Denmark; 4 Department of Biochemistry and Structural Biology, Lund UniversityLund, Sweden

**Keywords:** aquaporin-9, atherosclerosis, *branched chain ketoacid dehydrogenase*, metabolomics, *Nt5e*

## Abstract

Deletion of the glycerol channel aquaporin-9 (*Aqp9*) reduces postprandial blood glucose levels in leptin receptor–deficient (*db/db*) obese mice on a C57BL/6 × C57BLKS mixed genetic background. Furthermore, shRNA-mediated reduction of *Aqp9* expression reduces liver triacylglycerol (TAG) accumulation in a diet-induced rat model of obesity. The aim of this study was to investigate metabolic effects of *Aqp9* deletion in coisogenic *db/db* mice of the C57BL/6 background. *Aqp9*^*wt*^ *db/db* and *Aqp9*^*−/−*^ *db/db* mice did not differ in body weight and liver TAG contents. On the C57BL/6 genetic background, we observed elevated plasma glucose in *Aqp9*^*−/−*^ *db/db* mice (+1.1 mmol/L, life-time average), while plasma insulin concentration was reduced at the time of death. Glucose levels changed similarly in pentobarbital anesthetized, glucagon challenged *Aqp9*^*wt*^ *db/db* and *Aqp9*^*−/−*^ *db/db* mice. Liver transcriptional profiling did not detect differential gene expression between genotypes. Metabolite profiling revealed a sex independent increase in plasma glycerol (+55%) and glucose (+24%), and reduction in threonate (all at *q *<* *0.1) in *Aqp9*^*−/−*^ *db/db* mice compared to controls. Metabolite profiling thus confirms a role of AQP9 in glycerol metabolism of obese C57BL/6 *db/db* mice. In this animal model of obesity *Aqp9* gene deletion elevates plasma glucose and does not alleviate hepatosteatosis.

## Introduction

Aquaporin-9 (AQP9), a member of the major intrinsic protein family of transmembrane channels is permeable to glycerol, urea, and some other small, neutral solutes (Tsukaguchi et al. 1998). Expression has been demonstrated primarily in hepatocytes, along sinusoidal membranes as well as in a few other cell types (Rojek et al. 2008). In mice, *Aqp9* gene deletion results in elevated plasma glycerol levels (Rojek et al. 2007). Experiments in primary hepatocytes and perfused livers have demonstrated that AQP9 is essential for efficient glycerol gluconeogenesis (Jelen et al. 2011). Consistently, AQP9 expression is enhanced by prolonged fasting (Kuriyama et al. 2002; Calamita et al. 2012), whereby elevation in protein levels has been found specifically in male mice (Lebeck et al. 2012, 2015). Furthermore, *Aqp9* gene deletion has resulted in lower 4-h fasting blood glucose values in a leptin receptor–deficient *db/db* mouse model of type 2 diabetes (Rojek et al. 2007). The latter experiments were conducted in mice on a mixed C57BL/6 × C57BLKS genetic background (Rojek et al. 2007). These results have confirmed findings from in vitro and ex vivo experiments, but do not rule out that reduced blood glucose levels in *Aqp9*^*−/−*^ *db/db* may be the result of genetic linkage between *Aqp9* and a hypothetic modifier gene that differs between the C57BL/6 and the C57BLKS strains.

Besides its function in glycerol gluconeogenesis, hepatocyte glycerol uptake through AQP9 may supplement triacylglycerol (TAG) synthesis (Lebeck et al. 2015). In agreement with this hypothesis, shRNA-mediated silencing of *Aqp9* has alleviated nonalcoholic fatty liver disease (NAFLD) in a diet-induced rat model of obesity (Cai et al. 2013). In this study we have conducted a comprehensive phenotypic analysis of coisogenic *Aqp9*^*wt*^ and *Aqp9*^*−/−*^ mice, aiming for enhanced understanding of the metabolic role of AQP9 in mice on the C57BL/6 *db/db* genetic background, including its proposed role in TAG synthesis and NAFLD.

## Materials and Methods

### Animals

Experimental animals were housed in a specified pathogen-free facility at the Department of Biology, Lund University, Sweden. *Aqp9*^*−/−*^ C57BL/6 mice have been described previously (Rojek et al. 2007). B6.BLKS(D)-Leprdb/J mice, a backcross of the *Leprdb* allele from BLKS into the C57BL/6 background, performed at Jackson laboratories, were obtained from Charles River Laboratories, Germany, and bred with *Aqp9*^*−/−*^ C57BL/6 mice to obtain double mutants. Genotyping was performed as previously described in detail (Rojek et al. 2007; The Jackson Laboratory protocols database). Body weight and 4 h fasting blood glucose (ACCU-CHEK Aviva glucose meter from punctured tail vein) were determined weekly between 6 and 18 weeks of age. For the final 2 experimental weeks, mice were fed a specified content control diet (Altromin, Germany, #20000033). The diet was thawed 3–5 times per week from aliquots of one diet batch. All procedures were approved by the Malmö–Lund ethical committee for animal experiments.

### Sample collection and preparation

At 18 weeks of age mice were starved for 4 h, anesthetized (fentanyl/medetomidine/midazolam, 0.05/5/0.5 mg/kg body weight, intraperitoneal), decapitated, and exsanguinated into microvette 500 (Sarstedt) lithium–heparin tubes for plasma preparation. Following laparotomy, approximately 50 mg of liver tissue was excised quickly, weighed, briefly homogenized in 1 mL of ice-cold methanol/water (1/1, v/v), and frozen in liquid nitrogen (liver sample 1). Another liver aliquot was placed into TRIzol (Invitrogen, Carlsbad, CA), homogenized, and frozen in liquid nitrogen (liver sample 2). Plasma samples were subsequently frozen in liquid nitrogen.

### Hormone and lipid analysis

Liver sample 1 was thawed and the volume adjusted by methanol/water (1/1, v/v) to yield a tissue concentration of 50 mg/mL. The sample was then homogenized and metabolites and lipids were extracted as previously described in detail (Want et al. 2013). TAGs were analyzed utilizing a commercial kit (Sigma-Aldrich, Schelldorf, Germany, adipogenesis kit) from organic extract dried under a stream of nitrogen. Plasma insulin levels were determined by a mouse insulin ELISA (Mercodia, Sweden).

### Microarrays

RNA for microarray analysis was isolated from liver sample 2 utilizing the TRIzol® Plus RNA Purification System (Invitrogen). Hybridization to Affymetrix Mouse Gene 2.1 ST chips and data collection was performed by KFB Regensburg, Germany. Microarray data are available in the ArrayExpress database (http://www.ebi.ac.uk/arrayexpress) under accession number E-MTAB-3526. Data were processed using the oligo package in R/Bioconductor v3.1 (Gentleman et al. 2004). CEL files were background corrected using the RMA method and quantile normalized. Probesets with interquartile percentage (iqrPCT) greater than 0.1 were selected with genefilter v1.46.1. Probesets representing controls were excluded prior to further analysis. Differentially expressed genes were selected based on Student's *t*-test after correction for multiple comparisons using the Benjamini–Hochberg method (FDR < 0.1).

### Metabolite profiling

Fifty microliters of aqueous metabolite extract from liver sample 1 was vacuum centrifuged and metabolites were derivatized as previously described in detail (Danielsson et al. 2010). Plasma metabolites were extracted from 15 *μ*L plasma using a one-phase liquid extraction protocol, vacuum centrifuged, and derivatized as previously described in detail (Spégel et al. 2010; Spegel et al. 2013). Metabolites were analyzed on an Agilent 6890N gas chromatograph (Agilent, Atlanta, GA), equipped with a 30 m DB-5 msec Ultra Inert column (ID 250 *μ*m, phase thickness 0.25 *μ*m; Agilent), connected to a Leco Pegasus III TOFMS electron impact time of flight mass spectrometer (Leco Corp., St. Joseph, MI), as previously described (Danielsson et al. 2010; Spégel et al. 2010; Spegel et al. 2013). Data were acquired using Leco ChromaTof (Leco Corp.) and processed by hierarchical multivariate curve resolution in MATLAB 7.0 (Mathworks, Natick, MA) as previously described (Jonsson et al. 2006). Metabolites were normalized using a set of stable isotope labeled internal standards (Chorell et al. 2009) and identified by retention indexes, calculated from injection of a homologous alkane series, and mass spectra, using in house developed libraries.

The normalized data were further analyzed in Simca v13.0 (Umetrics AB, Umeå, Sweden). An orthogonal projections to latent structures discriminant analysis (OPLS-DA) model was built using the genotypes as classes. The model had four significant components, one predictive and three orthogonal. The proportion of explained variation in the X-matrix (R2X) and the Y-matrix (R2Y) were 0.581 and 0.88, respectively. The predictive ability based on sevenfold cross-validation (Q2) was 0.384. Details about experimental design as well as raw spectral data are available at the Metabolights database (http://www.ebi.ac.uk/metabolights; accession number MTBLS219).

### Glucagon challenge

Mice at 18–20 weeks of age were starved for 16 h, from 17:00 h until 9:00 h, before intraperitoneal injection with pentobarbital (90 mg/kg). Anesthetized mice were used to reduce stress-induced glucocorticoid influence on gluconeogenesis. Pentobarbital was chosen for its minor effects on glucose metabolism in rodents (Johansen et al. 1994). Blood glucose was measured from the tail tip before and 15, 30, 60, and 90 min after a single subcutaneous glucagon injection (0.1 mg/kg body weight, in normal saline; Novo Nordisk). Mice were kept on isothermal pads during the procedure.

Statistical analyses were performed as indicated in the text and in figure legends, utilizing Prism 5 (Graphpad, La Jolla, CA) and Simca v13.0 (Umetrics).

## Results

**Figure 1:**
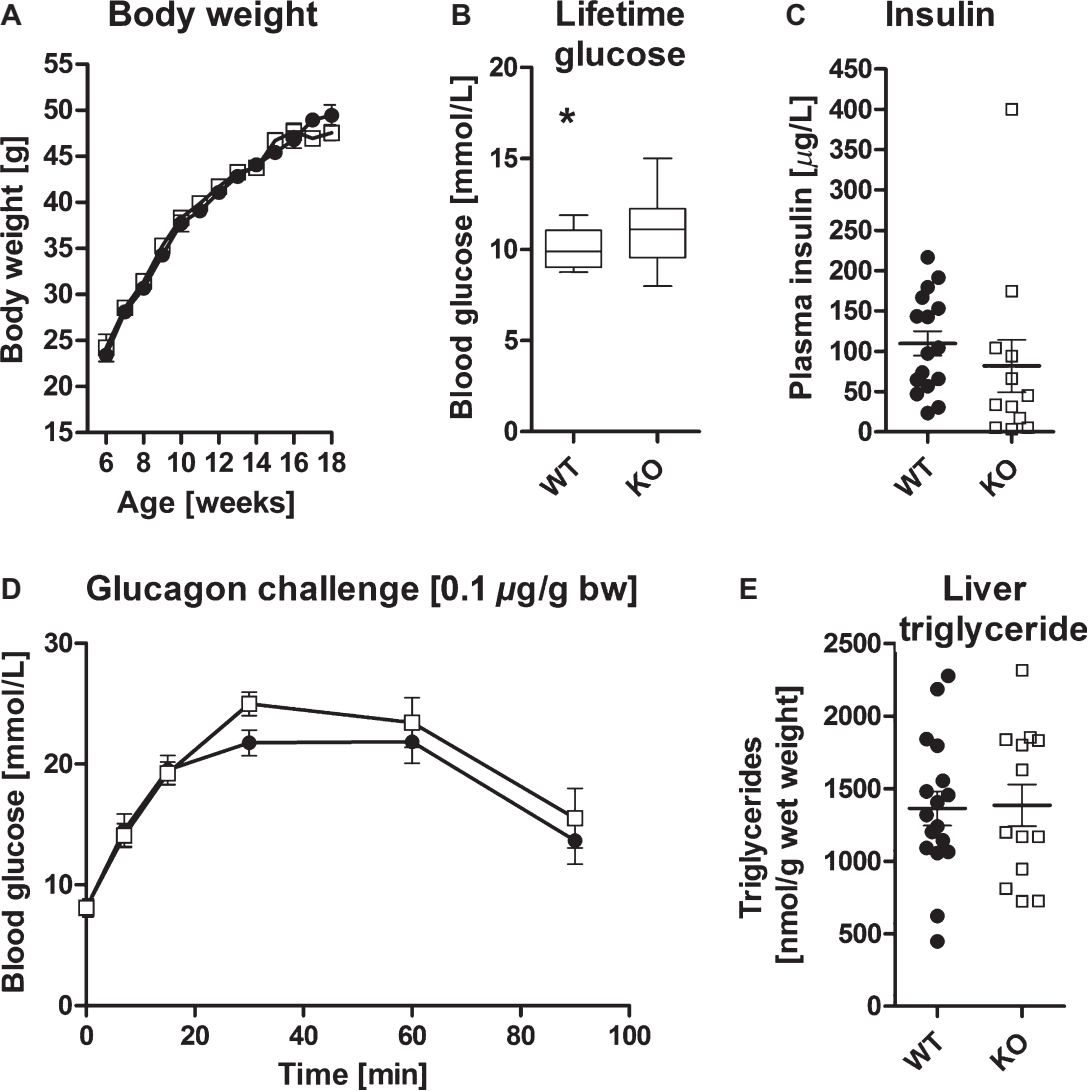
Phenotypic analysis of *Aqp9*^*wt*^ and *Aqp9*^*−/−*^ C57BL/6 *db/db* mice. (A) Body weight increased similarly in *Aqp9*^*wt*^ and *Aqp9*^*−/−*^ mice (mean, SEM). (B) Cumulative analysis of life-time blood glucose levels revealed elevated blood glucose in *Aqp9*^*−/−*^ mice (*P *=* *0.02; whiskers indicate min–max). (C) Plasma insulin data as shown were not normally distributed for *AQP9*^*−/−*^ mice (D'Agostino & Pearson omnibus normality test, *P *<* *0.0001). Accordingly, log_2_-transformed data analysis showed reduced plasma insulin in *AQP9*^*−/−*^ mice (*P *=* *0.03, mean, SEM). (D) Glucagon challenge induced similar elevation in blood glucose in both genotypes (mean, SEM). (E) Liver TAG content did not differ between genotypes. *Aqp9*^*wt*^ (WT), solid discs; *Aqp9*^*−/−*^ (KO), open squares. Differences between groups were assessed by two-way ANOVA (A, D) or Student's *t*-test (B, C, E), for *n *=* *25 (KO) and *n *=* *46 (WT) in (A, B), *n *=* *12 (KO) and *n *=* *16 (WT) in (C), *n *=* *11 (KO) and *n *=* *12 (WT) in (D), and *n *=* *13 (KO) and *n *=* *17 (WT) in (E).

Two groups of animals were analyzed in the current study: *Aqp9*^*+/+*^ and *Aqp9*^*+/−*^ were collectively considered wild type with regard to AQP9 protein function *(Aqp9*^*wt*^*)* and compared to *Aqp9*^*−/−*^ mice. No statistically significant effects of heterozygosity were observed in this study (e.g., relative plasma glycerol in metabolite profiling data described below, *Aqp9*^*+/+*^ – 100%, *n *=* *4, vs. *Aqp9*^*+/−*^ – 113%, *n *=* *12, Student's *t*-test, *P *=* *0.66). In order to investigate the role of AQP9 in coisogenic C57BL/6 *db/db* mice we crossed the original C57BL/6 *Aqp9*^*−/−*^ strain (Rojek et al. 2007) with C57BL/6 *db/db* mice and monitored body weight and blood glucose of F2 generation mice, between 6 and 18 weeks of age. Body weight developed similarly in both groups of animals (Fig. 1A). Fasted blood glucose levels tended to be higher in *Aqp9*^*−/−*^ than in *Aqp9*^*wt*^ mice on the C57BL/6 *db/db* genetic background (Fig. 1B), which contrasts previous observations in C57BL/6 × C57BLKS mice (Rojek et al. 2007). Consistent with known hyperinsulemia in *db/db* mice, plasma insulin was high and variable in both groups of mice, showing unequal data distribution between genotypes with no normal data distribution in *AQP9*^*−/−*^ mice (Fig. 1C). Analysis by *t*-test was thus conducted on log_2_-transformed data, which identified lower plasma insulin in *Aqp9*^*−/−*^ mice (*P = *0.03; Fig. 1C).

Glycerol gluconeogenesis is physiologically most important after prolonged starvation (Calamita et al. 2012), when glycogen carbohydrate reserves have been depleted. We therefore exposed obese *Aqp9*^*wt*^ and *Aqp9*^*−/−*^ mice to 16 h of starvation, before injection with glucagon to temporarily elevate hepatic glucose output. Blood glucose changed similarly in both genotypes indicating similar hepatic glucagon sensitivity (Fig. 1D).

Recent studies have described reduced hepatosteatosis in a rat model of diet-induced obesity (DIO) when *Aqp9* expression was reduced by shRNA (Cai et al. 2013). Thus, we tested the effect of *Aqp9* deletion on triglyceride levels in liver tissue of C57BL/6 *db/db* mice (Fig. 1E). Liver TAG content did not differ between *Aqp9*^*wt*^ and *Aqp9*^*−/−*^ mice (Fig. 1E).

In order to comprehensively characterize phenotypic effects of *Aqp9* deletion in C57BL/6 *db/db* mice we conducted microarray gene expression profiling of liver tissue as well as GC-MS metabolic profiling of liver tissue and blood plasma preparations. Univariate analysis of microarray gene expression revealed differential expression of only two genes between *Aqp9*^*wt*^ and *Aqp9*^*−/−*^ mice (FDR < 0.1): expression of *Aqp9* as well as *cytochrome b5 reductase 4* (*Cyb5r4*) were lower in *Aqp9*^*−/−*^ mice (23% and 72% of *Aqp9*^*wt*^, respectively). Microarray signal intensity for *Cyb5r4* was, however, low in liver tissue preparations of both genotypes, and *Cyb5r4* function was thus not further investigated.

**Figure 2:**
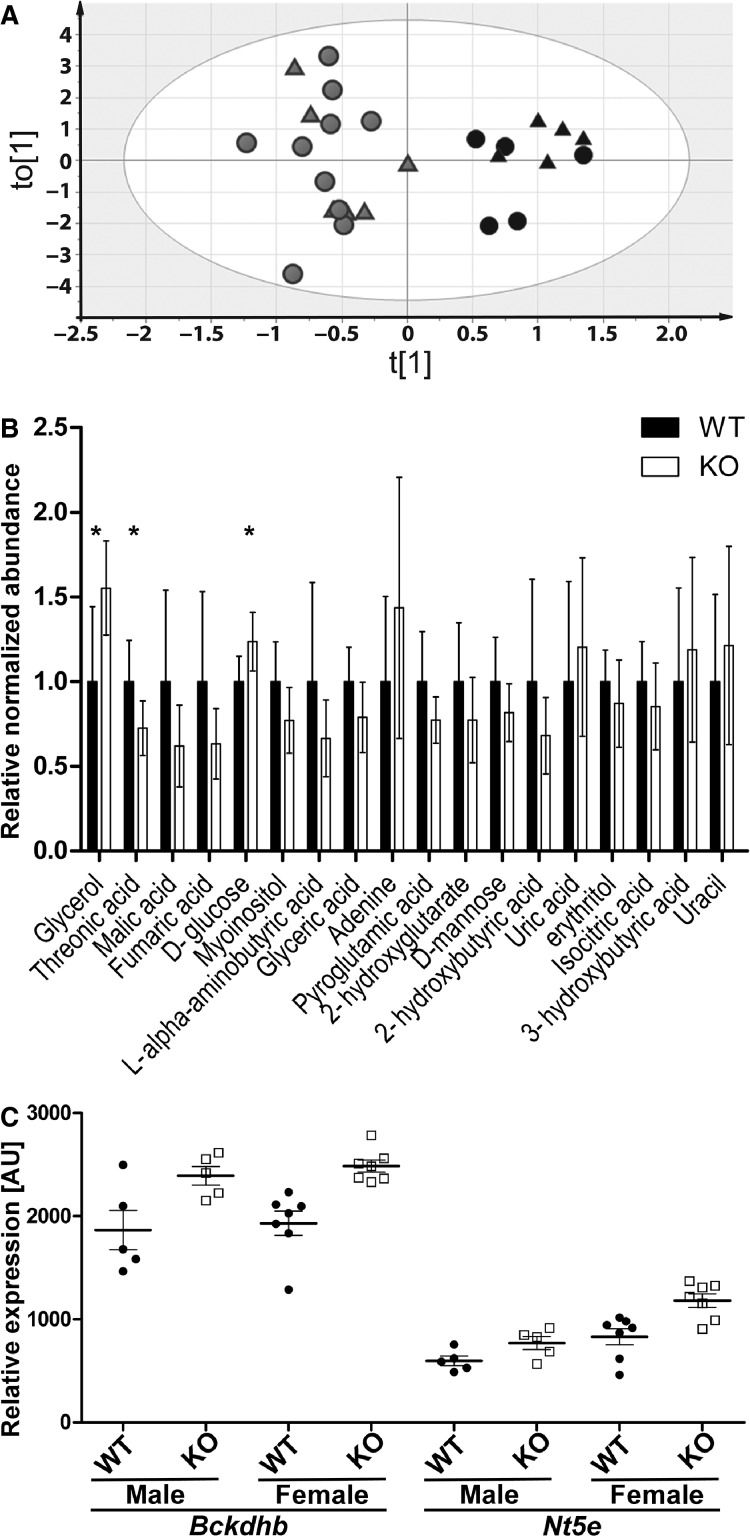
Analysis of GC-MS and microarray gene expression profiling of C57BL/6 *db/db Aqp9*^*wt*^ and *Aqp9*^*−/−*^ mice. (A) OPLS-DA facilitates plasma sample classification by genotype, indicating systematic differences in plasma metabolite profile between *Aqp9*^*−/−*^ and *Aqp9*^*wt*^ mice. Black = *Aqp9*^*−/−*^; gray = *Aqp9*^*wt*^; circle = male; triangle = female. (B) Relative abundance of plasma metabolites of OPLS-DA VIP score >1 in *Aqp9*^*wt*^ (WT) and *Aqp9*^*−/−*^ (KO) mice. VIP scores are sorted from left-highest to right-lowest. Metabolites with FDR < 0.1 in univariate statistics (*). (C) Relative gene expression of *Bckdhb* and *Nt5e* in *Aqp9*^*wt*^ (WT) and *Aqp9*^*−/−*^ (KO) mice, *t*-test: *Bckdhb P *=* *0.0355 (male), *P *=* *0.0036 (female); *Nt5e P *=* *0.0599 (male), *P *=* *0.0113 (female), FDR > 0.1.

Finally, we investigated alterations in circulating and liver metabolites by GC/MS. To this end, we determined relative levels of 61 and 84 metabolites in plasma and liver samples, respectively. Plasma concentrations of glucose, glycerol, and threonate differed between genotypes (FDR < 0.1; Data S1). Furthermore, OPLS-DA allowed classification of *Aqp9*^*wt*^ and *Aqp9*^*−/−*^ samples (Fig. 2A). Changes in metabolite levels underlying the observed clustering were investigated in the corresponding VIP plot. Thereby, in addition to the three metabolites identified by univariate analysis, two TCA cycle intermediates, fumarate and malate, were ranked highest by OPLS-DA VIP score (both VIP score 1.8; Fig. 2B).

Further inspection of these five metabolites revealed that average plasma glycerol and glucose concentrations were higher, and threonic acid, fumarate, and malate were lower in *Aqp9*^*−/−*^ mice of both genders. In contrast neither univariate analysis (FDR < 0.1) nor OPLS-DA identified differences between metabolite profiles of *Aqp9*^*wt*^ and *Aqp9*^*−/−*^ liver preparations. We note, however, that in agreement with plasma glucose values, average glucose-6-phosphate (1.6-fold), fructose-1-phosphate and 6-phosphate (1.5-fold), and glucose (1.2-fold) were elevated in liver samples of *Aqp9*^*−/−*^ (nominal *P *<* *0.05; Data S2).

In order to improve detection of moderate yet biologically meaningful differences between genotypes in gene expression and metabolite profiling we constructed gene–compound metabolic networks, utilizing Metscape (GAO et al. 2010; Karnovsky et al. 2012) in Cytoscape (Cline et al. 2007). Plasma metabolites of VIP score >1 (Fig. 2B) were used as input and suggested three metabolic networks, an amino acid metabolism–TCA cycle–butanoate metabolism network, a nucleotide metabolism network, and a glycerol metabolism network. Lists of genes in these networks were compared to top-ranked, albeit at FDR > 0.1, differentially expressed genes in *Aqp9*^*wt*^ and *Aqp9*^*−/−*^ liver microarrays. This approach identified 5′-nucleotidase, ecto (*Nt5e*) as part of the nucleotide network involving the metabolites adenine and uracil. The expression of this transcript appeared elevated in both male and female KO mice (Fig. 2C). Upon further inspection, we noticed that expression of the transcript *Bckdhb*, encoding the catalytic subunit of branched chain ketoacid dehydrogenase appeared also elevated in both male and female *Aqp9*^*−/−*^ mice (Fig. 2C). Branched chain ketoacid dehydrogenase was not part of the Metscape amino acid–TCA cycle–butanoate network. However, the enzyme is known to efficiently catalyze the conversion of 2-oxobutanoate to propanoyl-CoA (Jones and Yeaman 1986), which is an important substrate in this network.

## Discussion

*Aqp9* gene deletion effectively reduces postprandial blood glucose in a mouse model of type 2 diabetes (Rojek et al. 2007). In that study, leptin receptor–deficient *db/db* mice of mixed genetic background, C57BL/6 × C57BLKS, were used. Importantly, these genetic backgrounds differ significantly in their diabetic phenotype and disease progression: C57BLKS *db/db* mice develop severe diabetes characterized by hyperphagia, obesity, hyperglycemia, transient hyperinsulemia, and progressive pancreatic islet failure. In contrast, C57BL/6 *db/db* mice are hyperphagic, but display a more moderate phenotype that is explained by marked pancreatic hypertrophy partially compensating for insulin resistance (Hummel et al. 1972). Thus, C57BLKS *db/db* mice may be viewed as a model of late stage human type 2 diabetes, while the phenotype of C57BL/6 mice resembles an early diabetic or prediabetic, obese, insulin-resistant pathology.

Given the fundamental differences in these two mouse models, we decided to investigate the impact of *Aqp9* gene deletion in C57BL/6 *db/db* mice. We observed a moderate increase in blood glucose in these mice, contrasting the marked decrease in postprandial blood glucose observed in C57BLKS×C57BL/6 *db/db* mice (Rojek et al. 2007). A probable explanation for this unanticipated observation is divergent insulin control over hepatic glucose production between these mouse strains. The role of insulin in the regulation of hepatic glucose output is well established and affects both gluconeogenesis and glycogenolysis (Raddatz and Ramadori 2007). In healthy, postprandial animals hepatic glucose output is moderate. Similar balance, including moderate glycerol gluconeogenesis may be achieved in compensatory hyperinsulemic C57BL/6 *db/db* mice. On the other hand, hepatic glycerol gluconeogenesis may be constitutively elevated in C57BLKS mice. Interestingly, glucagon secretion is enhanced in pancreatic islet cells isolated from C57BLKS mice (Leiter et al. 1979). Constitutively elevated glucagon would likely result in elevated gluconeogenesis.

In the mixed genetic background hepatic glucose output is affected by random distribution of modifier genes in the genetic background. We can currently not rule out the possibility of genetic linkage between *Aqp9* and a putative modifier gene present in C57BLKS mice that may have accounted for reduced blood glucose levels in *Aqp9*^*−/−*^ mice on the mixed genetic background.

**Figure 3:**
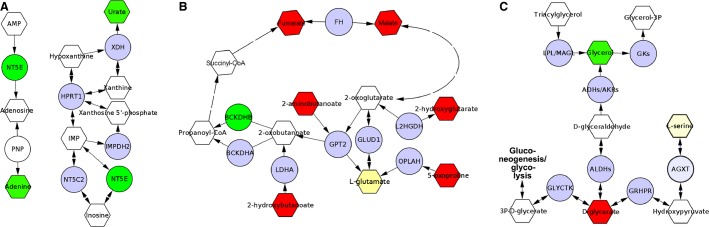
Networks constructed based on Metscape analysis including plasma metabolites of VIP score >1 and the transcripts *Nt5e* and *Bckdhb*. Up-in-*Aqp9*^*−/−*^, green; down-in-*Aqp9*^*−/−*^, red; unchanged transcripts, blue; unchanged metabolites, yellow; undetected, white.

We did not find any signs of changes in hepatic steatosis, contradicting a recent finding of reduced NAFLD in lentivirus-associated *Aqp9* RNA interference treated high-fat diet-fed Sprague Dawley rats (Cai et al. 2013). However, differences in the model and species used may account for this discrepancy. Comprehensive analyses of liver gene expression and liver as well as plasma metabolite levels did not reveal any strong effects of *Aqp9* gene deletion on all measured parameters. However, elevated plasma glycerol levels indicated that AQP9 function is reduced in these mice. Moderately elevated plasma glucose concentration in *Aqp9*^*−/−*^ mice, established with two independent methods, may thus reflect a compensatory shift in glucose metabolism presumably due to altered hormonal control. Average plasma insulin was indeed lower in the analyzed *Aqp9*^*−/−*^ mice even though data varied considerably between individual animals. This observation suggests reduced compensatory pancreatic hypertrophy in *AQP9*^*−/−*^ mice, possibly as a result of abolished hepatic glycerol gluconeogenesis (Jelen et al. 2011). Further putative compensatory adaptations were detected, including changes in adenine, uracil, malate, fumarate, and glycerates. Hypothetical pathway connections for these substances, including placement of two differentially expressed genes within the microarray data set, *Nt5e* as well as *Bckdhb* based on Metscape analysis are depicted in Figure 3. Based on known activity on 2-oxobutanoate (Jones and Yeaman 1986), elevated *Bckdhb* expression may explain depletion of several observed metabolites from *Aqp9*^*−/−*^ mouse plasma.

*Nt5e* is a plasma membrane protein that catalyzes the conversion of extracellular nucleotides to membrane-permeable nucleosides. Interestingly functional mutations in *Nt5e* are the cause of an inherited form of severe atherosclerosis: arterial calcification due to deficiency of CD73 (St Hilaire et al. 2011). On the other hand, high *Aqp9* expression has been associated with atherosclerosis in mice and humans (Inouye et al. 2012). While the reason for elevated plasma adenine and *Nt5e* expression in *Aqp9*^*−/−*^ mice remains unclear, this observation provides an explanation for the correlation between *Aqp9* expression and the development of atherosclerosis.

## Conclusions

We find that *Aqp9* gene deletion results in opposite phenotypic changes regarding glucose metabolism in a mouse model of early stage diabetes, compared to a previous study utilizing a mouse model of late stage diabetes (Rojek et al. 2007). While observations in mice are not readily applicable to treatment of human diabetes, we believe it is important to consider these effects of genetic background in future research of glycerol gluconeogenesis in type 2 diabetes.

## Supporting Information

**Data S1.** FDR analysis of measured plasma metabolite abundances.

**Data S2**. FDR analysis of measured liver metabolite abundances.
